# Supplementary material for: Dynalign II: common secondary structure prediction for RNA homologs with domain insertions
Source: Nucleic Acids Res. 2014 Nov 21;42(22):13939–48. doi: 10.1093/nar/gku1172 (PMC4267632; doi:10.1093/nar/gku1172)
Supplement: SUPPLEMENTARY DATA [file supp_gku1172_nar-02021-z-2014-File012.zip › manual/Developer/Javadocs.doc]

**RNAstructure**

**Developer's Manual**

**Javadocs**

**Version 1.0: 09/28/2012**

Creation of Javadocs for the RNAstructure repository occurs with a single script in the RNAstructure/manual/javadoc directory called javadocScript.

This script uses the javadoc tool (which is in the same directory as the java executable) to create a single unified group of Javadocs.

The comments folder inside the javadoc folder contains a group of HTML pages that are used to add additional information to the overview pages for individual Java packages. The HTML pages are named after the package they describe. For example, DrawingSource.html describes the package RNAstructure_java_drawing.source, which is in RNAstructure/RNAstructure_java_drawing/source. All the HTML page names reflect both the package names and the directory structure names for the Java code.

The HTML pages are temporarily copied into the package folder they represent and renamed package.html, then deleted after the Javadoc is made. This is necessary because the javadoc tool won't add extra information to a package page unless it's in a file called package.html. The copying, renaming, and deleting procedure keeps these comments, which are unique to the Javadocs, from getting mixed together unnecessarily with the Java source code.

To create Javadocs, all that is necessary is to go into the RNAstructure/manual/javadoc directory and execute javadocScript without any parameters. When the Javadocs are created, they will overwrite any existing Javadocs in the javadoc directory.
